# A systematic review and meta-analysis on abnormal posturing among brain injury patients

**DOI:** 10.1055/s-0044-1785689

**Published:** 2024-04-23

**Authors:** Yuyu Wei, Yan Cui, Xiaojun Pang, Weijie Wang

**Affiliations:** 1 Hangzhou Red Cross Hospital, Department of Neurosurgery, Hangzhou, Zhejiang, People's Republic of China.; 2 Affiliated Hospital of North Sichuan Medical College, Department of Emergency, Shunqing, Sichuan, People's Republic of China.; 3 Shandong University, The People's Hospital of Zhaoyuan City, Department of Neurosurgery, Zhaoyuan City, Shandong, People's Republic of China.

**Keywords:** Decerebrate State, Brain Injuries, Systematic Review, Brain Injuries, Estado de Descerebração, Lesões Encefálicas, Revisão Sistemática, Lesões Encefálicas

## Abstract

**Background** Abnormal motor posturing (AMP), exhibiting as decorticate, decerebrate, or opisthotonos, is regularly noticed among children and adults.

**Objective** This systematic review and meta-analysis examined the risk factors and outcome of posturing among severe head and brain injury subjects.

**Methods** Based on the inclusion and exclusion criteria and using MeSH terms: “decerebrate posturing”, “opisthotonic posturing”, “brain injury”, and/or “cerebral injury” articles were searched on Scopus, PubMed, Science Direct, and google scholar databases. Observational studies, case series, and case reports were included.

**Results** A total of 1953 studies were retrieved initially, and based on the selection criteria, 20 studies were finally selected for review and were analyzed for meta-analysis based on the mortality between the hematomas. The functional outcomes of this study are the risk factors, mortality rate and Glasgow Outcome Scale. Decerebrative patients were higher among the studies related to head injury surgeries. Males were mainly treated for decerebrate postures compared with the female subjects. Extradural hematoma and acute subdural hematoma with cerebral contusion were quite common in the surgical mass lesions.

**Conclusion** The findings reported that the lesion types, the operative procedures, and the age of the decerebrating patients with brain injuries are the significant prognostic factors determining the survival outcomes.

## Resumo

**Antecedentes** Postura motora anormal (AMP), exibindo-se como decorticada, descerebrada ou opistótono, é regularmente observada entre crianças e adultos.

**Objetivo** Esta revisão sistemática e metanálise examinou os fatores de risco e os resultados da postura entre indivíduos com lesões graves na cabeça e no cérebro.

**Métodos** Com base nos critérios de inclusão e exclusão e usando termos MeSH: artigos sobre “postura descerebrada”, “postura opistótona”, “lesão cerebral” e/ou “lesão cerebral” foram pesquisados nas bases de dados Scopus, PubMed, Science Direct e Google Scholar. Foram incluídos estudos observacionais, séries de casos e relatos de casos.

**Resultados** Um total de 1.953 estudos foram recuperados inicialmente e, com base nos critérios de seleção, 20 estudos foram finalmente selecionados para revisão e analisados para metanálise com base na mortalidade entre os hematomas. Os resultados funcionais deste estudo são os fatores de risco, taxa de mortalidade e Escala de Resultados de Glasgow. Os pacientes descerebrados foram maiores entre os estudos relacionados a cirurgias de traumatismo cranioencefálico. Os homens foram tratados principalmente para posturas descerebradas em comparação com as mulheres. Hematoma extradural e hematoma subdural agudo com contusão cerebral foram bastante comuns nas lesões de massa cirúrgica.

**Conclusão** Os achados relataram que os tipos de lesões, os procedimentos operatórios e a idade dos pacientes descerebrados com lesões cerebrais são os fatores prognósticos significativos que determinam os resultados de sobrevivência.

## INTRODUCTION

Abnormal motor posturing (AMP) includes decorticate and decerebrate postures response usually to noxious stimuli. The AMP is classified as decorticate and decerebrate postures in conventional movements of the trunk extension and edges with an increased muscular tone showing sign of brain injury. 1 2 Subjects with cerebral malaria, seizures, and other acute encephalopathies are commonly studied and associated with AMP. 3

Supratentorial and infratentorial lesions may cause both postures and are involved marginally in brainstem injury. 4 Decerebrate posturing might develop from brainstem lesions, whereas the decorticate posture regularly follows lesions at the cerebral cortex level. 5 Compared with decerebrate posturing, decorticate posture more stereotypically requires an injury more rostral and is considered the red nucleus at the intercollicular level in the middle of the brain. 6

Differentiating extradural hematoma (EDH) from subdural hematoma (SDH) in the head is typically forthright. Compared with EDH, SDHs are more common and identifiable with distinguishing features. 7 Intracerebral hemorrhage (ICH) in brain injury results in high morbidity and mortality. 8 Traumatic brain injury (TBI) is caused by a sudden, external, physical assault damages in the brain. A study estimated that each year, worldwide, there could be 69 million TBIs and 7.95% are considered as severe as the patients with a Glasgow Coma Scale (GCS) of 8 or less. 9 The GCS is the most substantial factor in prognosticating outcomes in brain injury, and the most specific one is the motor response pattern. 10 11 After a head injury, decerebrate rigidity is an important prognostic indicator of brain stem damages or compression secondary to tentorial herniation.

The mortality rate could increase from 33% to 70% for severe head injury (GCS <8) when the patient indicates signs of decerebration. 2 12 To achieve better results and avoid the conditions associated with high morbidity and mortality, prompt diagnosis, treatment, and proper management planning are needed. This study reviews the management and assessment of decorticate and decerebrate posturing among brain injury subjects and highlights the role of the prognosticators of outcomes of head and brain injury using meta-analysis.

## METHODS

This study follows Preferred Reporting Items for Systematic Reviews and Meta-Analyses (PRISMA) guidelines. 13 Based on criteria and eligibility, the articles were retrieved from the various electronic databases and were included for analysis.

### Literature search

PubMed, Scopus, Google Scholar, Science Direct, and Embase are the five databases used to screen the articles until March 2022. The keywords considered for the search were “abnormal posturing”, “decorticate posturing”, “decerebrate posturing”, “flexor posturing”, “posturing”, “opisthotonos”, “hematoma”, “brain injury”, “head injury”, “brain trauma”, and “skull fracture”. Using the Boolean operators: “AND”, “OR”, and “NOT,” the MESH terms were used to retrieve the articles.

### Eligibility criteria

The literature searches were limited based on our inclusion and exclusion criteria. Intervention, case-control, case reports, case series, and cross-sectional studies dealing with brain injury and posturing were included. Studies with any one of the postures among head and brain injury subjects with no limits on age, gender, ethnicity, and regions were recruited. There were no limitations on the date of publication or the regions where the studies were performed. The participants in the study needed to have decerebrate or decorticate posture associated with a brain injury. Abstracts, reviews, animal studies, studies with insufficient protocol, and articles with required information missing were excluded from this study. In addition, the reference section from the included literature and relevant retrospective studies articles were also reviewed for additional relevant studies.

### Data extraction

Two independent reviewers examined and extracted the data from the selected studies. Mortality and survival rate, Glasgow Outcome Scale (GOS), radiological findings, Glasgow Coma Scale (GCS), neurological manifestation, and operative procedures are the brain injury-related variables. All the data were recorded in Rayyan systematic review and Zotero for further analysis.

### Statistical analysis

The pooled mortality rates of decerebrate patients between different types of intracranial hemorrhage (EDH, SDH, and ICH) were evaluated using odds ratio (OR) values and the corresponding 95% confidence intervals (CI). Rayyan systematic review and Zotero software were used for systematic review analysis. The effect of the model selected depends upon the level of heterogeneity ( *I ^2^* ). Heterogeneity between studies was analyzed using the *I ^2^* statistic and Q-test, whereby *p* < 0.10 and *I ^2^* > 50% indicates high heterogeneity, and a random-effect model was applied for meta-analysis. Publication bias was visually identified using funnel plots, and any asymmetric plot suggests a possible publication bias. All data analysis was performed using Review Manager (“Version 5.3. Copenhagen: The Nordic Cochrane Centre, the Cochrane Collaboration, 2014”). The statistical significance was defined as a p-value of less than 0.05. We have assessed the risk of bias using the ROBINS-I in robvis tool ( https://mcguinlu.shinyapps.io/robvis/ ).

## RESULTS

### Study selection

**Figure 1:**
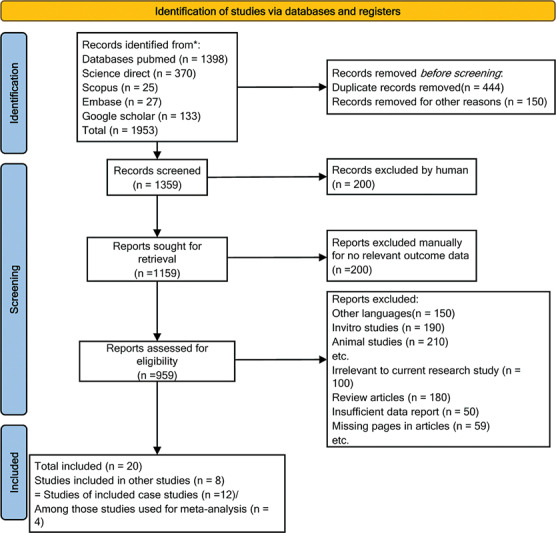
Prisma flow chart of the selected studies.

A total of 1953 articles were identified from the five literature databases. Among those, 444 duplicates were removed, and 150 articles were excluded based on the titles and abstracts. The leftover articles were screened and excluded based on the inclusion and exclusion criteria. Of 959 articles, 939 were excluded based on lack of information, irrelevant, animal studies, other languages, and review articles. Finally, the literature search from the five databases yielded 20 studies 5 14 15 16 17 18 19 20 21 22 23 24 25 26 27 28 29 30 31 32 until March 2022 ( Figure 1 ). The study characteristics of the selected studies are shown in Table 1 . Out of 20 articles, most of the studies were from the United States (45%), followed by Germany (10%), India (10%), and South Korea (10%), and the least, among Italy (5%), Kenya (5%), Canada (5%), Brazil (5%), and South Africa (5%). Most of the studies were: Case reports (12; 60%), and the rest were prospective and retrospective studies (40%).

**Table 1:** Study characteristics of the selected studies

No	Author, Year, Reference	Region	Sample size	Gender	Study	Age	Case subjects	Mortality Rate	Glasgow Outcome Scale (GOS)	Glasgow Coma Scale (GCS)
1.	Scarcella, 1962 14	USA	6	Case1= M Case2= M Case3= M Case4= F Case5= F Case6= M	CS	Case1= 5 Case2= 20 Case3= 5 Case4= 3 Case5= 18 Case6= 17	Decerebrate Rigidity in head injury	No deaths	NA	NA
2.	Celesia, 1963 15	Canada	1	Female	CR	10	Decerebrate attack in anoxic brain injury	No deaths	NA	NA
3.	Chatrian 1963 16	USA	1	Male	CR	59	Unresponsive decerebrate state in traumatic brain-stem infarct	Expired in respiratory and cardiac collapse after 14 days after the injury.	NA	NA
4.	Brendler, 1970 17	USA	16	M = 5, F = 4	Retrospective Study	Range = 14-67	Decerebrate rigidity in midbrain syndrome	Number of patients died: compressive lesions = 4, non-compressive lesions = 0	NA	NA
5.	Paul 1970 18	USA	52	M = 42 F = 10	Cross-sectional study	Mean = 30 range = 3-84	Decerebrate rigidity secondary to cranial trauma	Effect of surgical evacuation of intracranial hematoma on recovery from decerebration died: EDH = 3 (16-52 years age), SDH = 9(15-84 years age), ICH = 3(51-65 years age)	NA	NA
6.	North-Coombes 1972 19	South Africa	2	Case1: M Case2: M	CR	Case1: 50 Case2: 48	Decerebrate rigidity in subdural haematoma.	Case1: patient died	NA	NA
7.	Bricolo, 1977 20	Italy	800	NA	Compre- hensive Study	DP = 3 months to 86 years Non-DP-36	Decerebrate rigidity in acute head injury caused by traffic accidents	Motor signs outcome: decerebrate rigidity = (case No. 317; deaths No. 228 Full = (case No. 109; deaths No. 92	NA	NA
8.	Dong Hyun, 1977 21	South Korea	42	M = 33 F = 9	Cross-sectional study	10-70 years old	Decerebrate rigidity in head injury	Decerebrate patients 65% with direct damage to the brain stem supratentorial hematoma 52%	NA	NA
9.	Davis, 1983 22	USA	1	F	CR	28	Traumatic Decerebrate Rigidity in head injury	No deaths	NA	GCS = 4 on initial examination
10.	Klug, 1984 23	Germany	25	NA	Cross-sectional study	Mean(SD) = 35 range = 11-68	Severe head injury (15) and Acute vascular brain lesions (10)	NA	NA	Frequency of DR Episodes in Relation GCS, Cisternal Obstruction
11.	Pattisapu 1985 24	USA	1	M	CR	32	Unilateral brain stem lesion in a patient with traumatic decerebracy	Patient died	NA	NA
12.	Jabre, 1985 25	USA	1	Male	CR	20	Decerebrate posturing in head injury	No deaths	NA	NA
13.	Mahapatra, 1985 26	India	62	M = 52 F = 10	Retrospective study	Mean(SD) = 28.5(2.3) range = 2-73	Head-injured patients with bilateral decerebration	Neurological manifestation outcome: Pinpoint, non-reacting pupils= 2 died vestibuloocular reflex outcome: 8 patients with absent reflex died	NA	GCS = 4 on initial examination
14.	Briggs 1986 27	USA	1	M	CR	42	Decerebrate posturing after a head injury led to rhabdomyolysis and renal failure.	Patient died	NA	NA
15.	Damasceno, 1991 28	Brazil	1	Female	CR	27	Decerebrate rigidity with in anoxic-ischemic brain damage	No deaths	NA	NA
16.	Pranzatelli, 1991 29	USA	3	Case1: M Case2: M Case3: M	CS	Case1: 13 Case2: 7 Case3: 19	Decerebration with opisthotonus	No deaths	NA	NA
17.	Kiboi, 2009 30	Kenya	1	M	CR	46	Vertex epidural haematoma	No deaths	NA	On admission, the patient was unconscious with a Glasgow coma scale of 7/15 (motor 4, eye 2, verbal 1).
18.	Jung, 2013 31	South Korea	1	M	CR	51	Decerebrate Rigidity in anoxic brain damage	No deaths	NA	At admission, he revealed GCS score 7 (E4 V0 M3)
19.	Woischneck,2014 32	Germany	120	M = 82 F = 38	Prospective study	Median = 36, range = 2-86	Decerebrate posturing following traumatic brain injury	Decerebrate rigidity, no midbrain lesion = 19%; (b) without decerebrate rigidity, with midbrain lesion = 41%; (c) with decerebrate rigidity and with midbrain lesion = 33%; (d) with decerebrate rigidity, no midbrain lesion = 21%	Patients with a decerebrate response revealed significantly lower GOS scores than those without (p= 0.01). Detection of a brainstem lesion on MRI was correlated to the GOS (p < 0.0001). The correlation of midbrain lesions with the GOS was also significant (p < 0.0001).	NA
20.	Amit, 2015 33	India	72	M =57 F = 15	Retrospective study	>60 years old	Operated patients with decerebrate rigidity secondary cranial trauma	60 (83%) is the total mortality rate in the series.	Favorable outcome (GOS 4 and 5) in 14% (n = 10) of the patients	GCS 4 (M2) at the time of operation

Abbreviations: CS, Case series study; CR, Case report; F, Female; M, Male; NA, Not applicable.

### Clinical characteristics of the patients

Among the final retrieved articles, 1209 patients were assessed for this study. Based on the data available from the selected studies, most of them were males (286) compared with females (91). Those patients ranged between 3 to 86 years old. For most patients with any significant operable lesion, the Computed tomography (CT) scan was conducted in most of the studies for EDH, SDH, and cerebral contusions. Most studies have used craniotomies as the operative procedures to evacuate the routine trauma flaps for EDH, acute SDH, and cerebral contusions. After the surgery, steroids, mannitol, and intravenous solution were the common treatment given to the patients. Seizures, unilateral decerebrate rigidity, and decorticate rigidity are the common outcomes of the selected studies.

### Mortality outcomes

**Figure 2:**
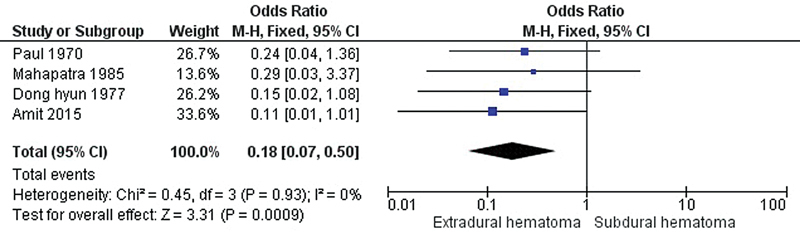
Forest plot of the odds ratio for mortality between decerebrate patients with EDH and SDH.

**Figure 3:**
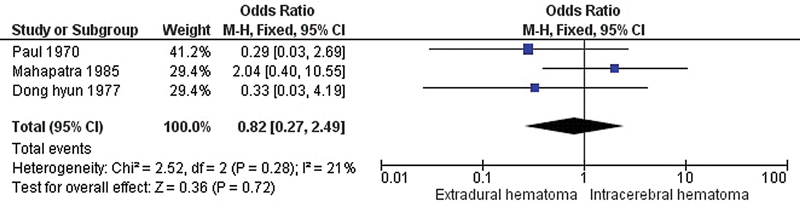
Forest plot of the odds ratio for mortality between decerebrate patients with EDH and ICH.

**Figure 4:**
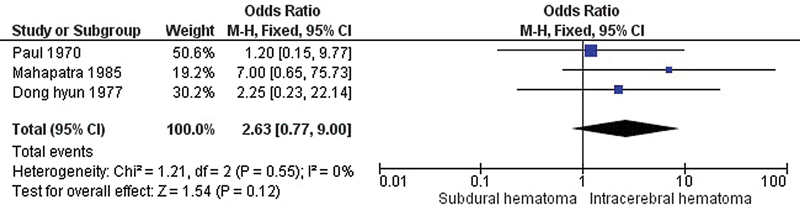
Forest plot of the odds ratio for mortality between decerebrate patients with SDH and ICH.

Based on the GCS scores, the comparison of EDH, SDH, and ICH was analyzed based on the mortality rates by injury severity. Hence, only four 16 24 26 32 studies were included for meta-analysis. Figure 2 shows a forest plot comparing the EDH and SDH groups. The results indicated that decerebrate patients with EDH have a greater mortality rate than SDH (pooled OR = 0.18, 95% CI = 0.07–0.50, *p* < 0.05). Figure 3 shows a forest plot comparing the EDH and intracerebral hematoma (ICH) groups. No significant difference was noticed between the mortality rate of decerebrate patients with EDH compared with ICH (pooled OR = 0.82, 95% CI = 0.27–2.49, *p* = 0.72). Figure 4 shows a forest plot comparing the SDH and ICH groups. The results show no significant difference between the mortality rate of decerebrate patients with SDH compared with ICH (pooled OR = 2.63, 95% CI = 0.77–9.00, *p* = 0.12).

### Funnel plot

**Figure 5:**
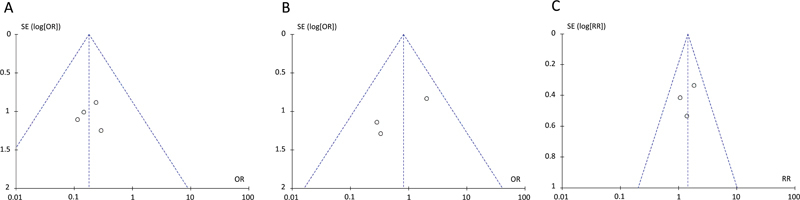
Funnel plot for publication bias of studies included in meta-analysis of mortality rates ( **A** ) between decerebrate patients with EDH and SDH, ( **B** ) between decerebrate patients with EDH and ICH and ( **C** ) between decerebrate patients with SDH and ICH.

The included studies showed low heterogeneity in overall mortality rates (I ^2^ < 50%, *p* >0.1). Therefore, the pooled ORs were calculated using a fixed-effect model analysis. A symmetry funnel plot of the included studies in all three models showed no publication bias ( Figure 5 ).

### Risk of bias

Supplementary Material ( https://www.arquivosdeneuropsiquiatria.org/wp-content/uploads/2024/02/ANP-2024.0009-Supplementary-Material-scaled.jpg ) shows the results of the risk of bias assessment generated using the ROBINS-1 tool for the observational studies alone, visualized with the use of robvis. 33 The studies 5 23 are the only two observational studies from the list of studies included as the other studies are not eligible to do the risk of bias due to the lack of information and case report studies.

## DISCUSSION

The AMP is a typical feature of severe head and brain injury and could be associated with the features of raised intracranial pressure and seizure recurrence. 34 A high mortality rate has been observed among AMP subjects with brain injury could raise the bar in treating the patients, whether or not they should be treated aggressively at an early stage. 20 35 An early prediction of survival and efficient outcomes is important for the physician to take appropriate and aggressive treatment. In this systematic review, we have analyzed the risk factors as the mortality and morbidity prediction and posturing outcome among severe head and brain injury subjects.

Most of the decerebrate patients followed by cranial trauma have survived and recovered from a decerebrate state in a reasonably functional state, which could be due to the evacuation of an intracranial hemorrhage. 14 15 17 25 28 In most survivors, the lack of serious neurological sequelae established the reversibility of brain stem compression.

The GCS score of 4 is considered a poor prognostic factor as an outcome of patients with a severe blunt head injury. 72% of mortality and 16% of worthy outcomes were noticed in the decerebrate patients. 20 Mahapatra et al. 1985 reported that 68% mortality and 18% recovery 26 had been noticed. Survival and recovery are based on the type of intracranial hematoma and the decerebrate state of the patients. Patients with EDH have a higher survival rate than SDH and ICH. The survival rate among subjects with cerebral contusions was poor but less than those with SDH. This has been well noticed in the reports 15 17 25 28 35 36 and is certainly related to the statement that patients with acute subdural and intracerebral hematomas have associated severe brain damage.

Age is the significant risk factor for survival after suffering traumatic decerebration. Scarcella et al. 14 reported that the recovery rate was less among subjects over 60 years compared with those under 60 years old. However, the cost and the management also play a vital role in survival among the patients after a severe head injury, as highlighted in many reports. 19 30 37

There were only two studies were selected for the risk of bias test as the other studies were mostly on case report study designs. Thus only two studies 5 32 were given an overall score of low which was determined by consensus agreement between the two co-authors and no other clear evidence for biases was found.

The effectiveness of medical treatment depends on the extent and the severity of the brain injury, the prognosis could vary among the patients with decerebrate posturing. 34 38 The recovery depends on the patients' complications, however, rehabilitation therapy could be helpful in regaining the function and improving their quality of life. 39 Brain injury patients with decerebrate or decorticate posture should be managed with psychological support, medications, assistive devices, and rehabilitation services. Studies have reported that infrastructural problems could increase the functional outcome and might decrease mortality, as interpreted in a study on severe head injury. 40

### Limitations of the present study

There are a few limitations that need to be accounted for in this study. The outcomes of predictive factors must be made with more attention. Furthermore, the records of follow-up of patients were not carried out. In addition, a critical factor that affects the outcome of decerebrate patients is the precise duration of decerebration 18 was not studied. The decerebrate rigidity takes place at a rate of 30–70% over the progression of severe TBI, MRI findings detecting the number of lesions in diverse areas of the brain also need to be recorded to determine the correlation between decerebrate posturing and lesions of the brain. The substantial heterogeneity among the patient profiles is a hallmark characteristic of TBI, hence it must be studied and analyzed in the study design.

In conclusion, the findings of this study show that poor prognosis and management in decerebrate patients have high mortality among the patients. Lesion types, age, and the duration of decerebration are the most significant prognostic factors that determine the outcome of surgery. Understanding the pathophysiology, management, prevention, and a strategy to overcome the barriers and knowledge gaps in treating brain injury patients with care is much needed to reduce the mortality rate.
